# Current Status of Drug Prescribing among Older Adults with Advanced-stage Cancer Receiving Home Medical Care in Japan: A Nationwide Study

**DOI:** 10.31662/jmaj.2025-0179

**Published:** 2025-09-26

**Authors:** Yukari Hattori, Shota Hamada, Masao Iwagami, Nobuo Sakata, Kiwami Kidana, Nanako Tamiya, Masahiro Akishita, Takashi Yamanaka

**Affiliations:** 1 Department of Home Care Medicine, Graduate School of Medicine, The University of Tokyo, Tokyo, Japan; 2 Research Department, Institute for Health Economics and Policy, Association for Health Economics Research and Social Insurance and Welfare, Tokyo, Japan; 3 Department of Health Services Research, Institute of Medicine, University of Tsukuba, Tsukuba, Japan; 4 Heisei Medical Welfare Research Institute, Tokyo, Japan; 5 Tokyo Metropolitan Institute for Geriatrics and Gerontology, Tokyo, Japan

**Keywords:** cancer, deprescribing, end-of-life, home healthcare, older adults, polypharmacy

## Abstract

**Introduction:**

Balancing preventive and symptomatic medications is crucial to minimizing polypharmacy in older adults with limited life expectancy. This study examined end-of-life medications among older adults with advanced-stage cancer receiving home medical care.

**Methods:**

We conducted a retrospective cohort study using Japan’s national claims database between October 2017 and September 2019 and selected adults aged ≥75 years who received comprehensive home medical care services for patients with advanced-stage cancer. We compared prescriptions for preventive and symptomatic medications during two periods: (1) within 180 days before the first claim for the service issued (index date) and (2) within 180 days after the index date or until death.

**Results:**

Overall, the study included 8,463 participants, of whom 47% were women and 46% were aged ≥85 years. The median observation period after the index date was 57 days (interquartile range: 30-131 days). Among preventive drugs, antihypertensives were the most frequently prescribed before the index date (60.3%), followed by lipid-lowering drugs (25.4%) and antiplatelets (21.7%). Prescription of lipid-lowering drugs, vitamins, and drugs for osteoporosis decreased by approximately 70%-80% after the index date. In contrast, prescription of oral anticoagulants, antidiabetic drugs, and antidementia drugs declined by approximately 40%. Symptomatic drug prescriptions also decreased after the index date, except for opioids (from 42.2% to 51.9%).

**Conclusions:**

This is the first nationwide study to examine the prescriptions of preventive and symptomatic drugs among older adults with advanced-stage cancer receiving end-of-life home medical care.

## Introduction

The home is the preferred place of death for most older adults in Japan ^(1)^. Home medical care plays an important role in supporting older adults until their end-of-life stage by providing medical care from a relatively stable phase to the end-of-life phase to achieve the goals of aging and dying. Population aging is accompanied by an increase in the number of older adults with cancer, and adults aged ≥75 years accounted for 45.4% of the patients with cancer in Japan in 2019 ^(2)^. Most patients with cancer prefer to stay at home at the end-of-life stage ^(3)^, and, from the perspective of the efficient use of hospital resources, the number of patients with cancer who receive home medical care will increase.

For patients with advanced-stage cancer, controlling various symptoms, including fatigue, weakness, appetite loss, urinary incontinence, asthenia, pain, constipation, and anxiety, is crucial ^(4), (5)^. In addition, older adults with cancer are more prone to geriatric syndromes, including hearing problems, urinary incontinence, falls, depression, and osteoporosis ^(6)^. For older adults with advanced-stage cancer, the goals of treatment and care should be re-evaluated and focused on maintaining quality of life (QOL).

During the medication optimization process, balancing preventive and symptomatic medications is important to avoid polypharmacy at the end-of-life stage. Some preventive drugs, which prevent the development of a disease or mitigate the progression, such as statins and antiplatelets, can be rationally deprescribed based on the principle of time to benefit ^(7), (8)^. Medications that control symptoms, defined as symptomatic drugs, may be added to control symptoms in patients approaching the end of life ^(9)^. A recent systematic review concluded that deprescribing interventions can improve medication appropriateness in older adults with life-limiting illnesses and a limited life expectancy ^(10)^.

Nevertheless, previous studies have shown that preventive drugs are often continued until the end-of-life stage. A nationwide study conducted in Sweden reported that preventive drugs, such as antihypertensives, antiplatelets, anticoagulants, statins, and oral antidiabetics, remained prescribed until the final weeks before death in older adults with solid tumors who died between 2007 and 2013 ^(11)^. Moreover, an observational study conducted in France showed that drugs for thrombosis prevention and antidiabetic drugs continued to be prescribed at the end-of-life stage in hospitalized patients receiving palliative care between 2010 and 2011 ^(12)^. Notably, several guidelines and tools were published over the past decade to support deprescribing in oncological palliative care ^(13)^ and older adults approaching the end of life ^(14)^. The OncPal guidelines, the deprescribing guidelines for patients with cancer with an estimated prognosis of ≤6 months ^(13)^, and Screening Tool of Older Persons Prescriptions in Frail adults with limited life expectancy (STOPPFrail), the list of medications considered for deprescribing for frail older adults with an estimated prognosis of ≤1 year ^(14)^, have been reported to be adequately performed ^(15)^.

Information on end-of-life home medical care among older adults with cancer remains scarce in Japan ^(16)^. Our previous report on medication use among older adults receiving home medical care suggested that the number of prescribed drugs remained unchanged; however, the use of potentially inappropriate medications, except for antipsychotics and diuretics, decreased between 2015 and 2019 ^(17)^. Another study examining medication changes in the last year of life found that a cancer diagnosis was associated with a decrease in the number of preventive drugs ^(18)^. However, these studies did not specifically focus on older adults with advanced-stage cancer in the last months of life, when discontinuing preventive drugs is typically prioritized. Thus, this study aimed to elucidate the status of end-of-life medications among older adults with advanced-stage cancer receiving home medical care.

## Materials and Methods

### Data source

This study was a retrospective cohort study using data from older adults aged ≥75 years from the National Database of Health Insurance Claims and Specific Health Checkups of Japan (NDB), which contained almost all the medical and pharmacy claims data across the country with universal health coverage ^(19)^. Generally, people aged ≥75 years in Japan are covered by the Late Elders’ Health Insurance system, managed by local governments. Because most older adults aged ≥75 years who received home medical care at the end of life were not expected to change their insurance system (e.g., due to transferring out of a municipality), “ID1” was used as the same patient identifier in the NDB, which was generated based on an individual’s insurance identification number, birth date, and sex.

### Study participants

We selected older adults aged ≥75 years who received comprehensive home medical care services for patients with advanced-stage cancer for the first time (index date; the date of the first claim for this service, with no previous claims for the service in the preceding 6 months) between October 2017 and September 2019. When patients who receive home medical care enter the end-of-life phase, the additional support plan called “comprehensive home medical care services for patients with advanced-stage cancer” can be implemented. Thus, they can receive medical and care support intensively throughout their final phase. For the baseline assessment before the index month, we included patients who received out-of-hospital medical services (including outpatient services by hospitals, clinics, or home medical care services) at least during both the 4-6 month period and the 1-3 month period before the index month. We excluded those who died or those who could not be tracked for at least 15 days because we focused on drug prescriptions after initiating comprehensive home medical care services for patients with advanced-stage cancer.

### Measurements

We evaluated the prevalence of drugs at two different time points: (1) within 180 days before the index date (baseline) and (2) within 180 days after the index date or until death. We studied as preventive drugs including antihypertensives, antiplatelets, oral anticoagulants, lipid-lowering drugs, antidiabetic drugs, drugs for osteoporosis, antigout drugs, antidementia drugs, calcium, iron, and vitamins. These drugs are generally considered ineffective or have questionable benefits in patients with limited life expectancies ^(11), (13), (18), (20), (21)^. As symptomatic drugs, we examined some frequently prescribed drugs, including acid suppressants, laxatives, analgesics, and hypnotics, based on our previous study in older adults with limited life expectancies receiving home medical care ^(18)^.

Referring to the previous study in Sweden ^(11)^, we evaluated the prevalence of drugs of interest before and after the index date and calculated the proportions of continuation and initiation. Continuation was defined as drugs that were used before the index date and after the index date. In contrast, initiation was defined as drugs that were not used in the baseline period and prescribed after the index date.

Age and sex were evaluated based on the index date. Diagnoses, including cancer and comorbidities, were assessed using International Classification of Diseases, Tenth Revision codes, and the use of regular home medical care services and hospitalizations were evaluated during the 6-month baseline period.

### Statistical analysis

Descriptive statistics were used to summarize the characteristics of the study participants and the prevalence of prescriptions before and after the index date. Analyses were conducted for all participants and according to three major cancer types (lung, colorectal, and gastric cancer). All analyses were performed using Stata version 14 software (Stata Corp., College Station, TX, USA).

## Results

### Baseline characteristics

Among 10,923 participants aged 75 years or older who received comprehensive home medical care services for patients with advanced-stage cancer, we selected the 10,398 participants who had claims for out-of-hospital medical services during the baseline period. Subsequently, we selected those with 15 days or longer follow-up, and a total of 8,463 participants were included in the further analysis.

Men or individuals aged 75-84 years accounted for more than half of the study participants (Table 1). The median (interquartile range [IQR]) observation period after the index date was 57 days (IQR: 30-131 days). Among these patients, 21.1% had lung cancer, followed by colorectal (19.2%), gastric (16.5%), pancreatic (10.5%), and prostate cancer (10.2%). The most common comorbidities were heart failure and diabetes mellitus, followed by ischemic heart disease, dementia, and asthma or chronic obstructive pulmonary disease.

**Table 1.** Baseline Characteristics (n = 8,463).

Characteristic		n (%)
Age (years)	75-79	2,061 (24.4)
	80-84	2,554 (30.2)
	85-89	2,217 (26.2)
	90-94	1,246 (14.7)
	≥95	385 (4.5)
Sex	Men	4,505 (53.2)
	Women	3,958 (46.8)
Diagnosis: cancer (ICD-10)	Solid	7,917 (93.6)
	Gastric (C16)	1,394 (16.5)
	Colorectal (C18-C20)	1,624 (19.2)
	Hepatobiliary (C22)	676 (8.0)
	Pancreas (C25)	892 (10.5)
	Lung (C34)	1,787 (21.1)
	Breast (C50)	420 (5.0)
	Female genital organs (C51-C58)	339 (4.0)
	Prostate (C61)	867 (10.2)
	Kidney and ureter (C64-C66, C68)	350 (4.1)
	Bladder (C67)	428 (5.1)
	Hematological	507 (6.0)
	Non-Hodgkin lymphoma (C82-C85)	297 (3.5)
	Multiple myeloma (C90)	108 (1.3)
	Unknown	110 (1.3)
Diagnosis: comorbidity (ICD-10)	Asthma or Chronic obstructive pulmonary disease (J43-J46)	2,193 (25.9)
	Dementia (F00-F03, G30)	2,229 (26.3)
	Depression (F31-F33)	1,409 (16.7)
	Diabetes mellitus (E10-E14)	3,712 (43.9)
	Heart failure (I50)	3,784 (44.7)
	Ischemic heart disease (I20-I25)	2,466 (29.1)
	Parkinson’s disease (G20)	251 (3.0)
	Stroke (I60-I64)	1,564 (18.5)
Home medical care services	Previous 6 months	5,406 (63.9)
Hospitalization	Previous 6 months	6,035 (71.3)
Geographic regions	Hokkaido/Tohoku	951 (11.2)
	Kanto	3,708 (43.8)
	Chubu	1,269 (15.0)
	Kansai	1,614 (19.1)
	Chugoku/Shikoku	442 (5.2)
	Kyushu/Okinawa	479 (5.7)

ICD-10: International Classification of Diseases, Tenth Revision.

More than half of the patients (63.9%) received home medical care services at least once within six months before the index date, and most patients (71.3%) were hospitalized before receiving home medical care services.

### Preventive drugs

Antihypertensives were most frequently prescribed among the preventive drugs before the index date (60.3%), followed by lipid-lowering drugs (predominantly statins; 25.4%) and antiplatelet drugs (21.7%) (Table 2).

**Table 2.** Prescribing of Preventive Drugs.

Drugs	Prevalence			Change	
	Before (%)	After (%)	Change (%)	Continuation (%)	Initiation (%)
Antihypertensives	60.3	30.4	−49.5	45.4	7.6
ACEI/ARB	35.0	13.2	−62.3	33.2	2.5
β blockers	12.6	7.7	−39.2	50.2	1.5
Calcium channel blockers	46.1	20.5	−55.6	39.1	4.6
Diuretics^a^	6.7	2.6	−61.6	29.4	<1.0
Others	3.3	1.3	−58.8	30.6	<1.0
Antiplatelets	21.7	10.8	−50.5	45.5	1.1
Aspirin	14.5	6.5	−54.9	41.9	<1.0
P2Y12 inhibitors	7.3	3.4	−53.6	41.8	<1.0
Others	4.2	2.0	−52.2	40.4	<1.0
Oral anticoagulants	10.7	6.4	−40.7	47.5	1.4
DOAC	8.2	5.2	−37.1	48.4	1.3
Warfarin	3.1	1.2	−59.7	31.8	<1.0
Lipid-lowering drugs	25.4	5.7	−77.5	21.0	<1.0
Statins	22.1	5.0	−77.6	20.9	<1.0
Antidiabetic drugs	18.4	10.5	−43.0	49.0	1.8
Non-insulin	17.3	8.9	−48.5	44.8	1.4
Insulin	4.7	2.9	−38.2	39.4	1.1
Drugs for osteoporosis	19.6	6.0	−69.5	26.3	1.0
Bisphosphonates	9.8	2.0	−79.4	16.5	<1.0
Vitamin D formulations	12.5	4.5	−64.3	30.3	<1.0
Antigout drugs	12.5	4.6	−63.0	30.9	<1.0
Antidementia drugs	11.7	6.6	−43.4	46.0	1.4
Acetylcholinesterase inhibitors	9.6	4.9	−48.5	42.8	<1.0
Memantine	3.7	2.5	−33.6	47.9	<1.0
Calcium	2.1	<1.0	NA	23.8	<1.0
Iron	18.0	9.8	−45.4	35.9	4.1
Vitamins (B, C, or multivitamins)	19.8	6.0	−69.6	21.7	2.2

ACEI: angiotensin-converting enzyme inhibitor; ARB: angiotensin II receptor blocker; DOAC: direct oral anticoagulant; NA: not applicable. ^a^Diuretics as drugs other than thiazides and selective aldosterone blockers (e.g., loop diuretics and potassium-sparing diuretics).

The relative decrease was the largest for lipid-lowering drugs (−77.5%), followed by vitamins (−69.6%) and drugs for osteoporosis (−69.5%). The smallest relative decreases were observed for oral anticoagulants (−40.7%), antidiabetic drugs (−43.0%), and antidementia drugs (−43.4%). Among the same therapeutic category, the changes in prescribing varied: for example, the decreases in prescribing of β blockers in antihypertensives, direct oral anticoagulants in oral anticoagulants, insulins in antidiabetic drugs, and memantine in antidementia drugs were smaller than other classes of drugs.

Most of preventive drugs prescribed after the index dates were continued and the initiation rates were generally low. Some drugs had higher initiation rates, including antihypertensives (7.6%), iron (4.1%), and vitamins (2.2%).

### Symptomatic drugs

Symptomatic drug prescriptions also decreased after the index date, except for opioids (Table 3). Analgesics were most frequently prescribed before the index date (78.7%), followed by laxatives (67.4%) and acid suppressants (65.5%).

**Table 3.** Prescribing of Symptomatic Drugs.

Drugs	Prevalence			Change	
	Before (%)	After (%)	Change (%)	Continuation (%)	Initiation (%)
Acid suppressants	65.5	47.4	−27.6	59.6	24.2
Proton pump inhibitors	58.2	43.9	−24.5	59.6	22.1
H_2_ receptor antagonists	13.9	4.3	−68.8	22.1	1.5
Laxatives	67.4	49.8	−26.1	57.4	34.1
Diuretics (non-antihypertensives)	30.1	24.6	−18.3	53.6	12.1
Loop	27.8	22.8	−18.0	52.1	11.5
MRA	12.8	10.5	−17.9	45.2	5.4
Analgesics	78.7	68.1	−13.5	72.8	50.8
Acetaminophen	53.1	30.1	−43.3	37.3	22.0
NSAIDs	49.5	25.9	−47.8	36.9	15.1
Opioids (oral and patch)	42.2	51.9	22.8	75.1	34.9
Opioids (oral)	39.9	43.4	9.0	65.2	29.0
Opioids (patch)	12.9	32.2	149.0	71.9	26.3
Hypnotics	33.3	25.7	−22.8	52.3	12.4
Benzodiazepines	17.8	13.2	−25.6	46.5	6.0
Z-drugs	15.8	10.1	−36.4	38.0	4.8
Newer drugs	8.2	7.4	−9.2	40.3	4.5

Oral drugs, otherwise specified. MRA: mineral corticoid receptor antagonist; NSAID: nonsteroidal anti-inflammatory drug.

The largest relative decrease was observed for acid suppressants (−27.6%), followed by laxatives (−26.1%), hypnotics (−22.8%), and diuretics (−18.3%). Within the same therapeutic category, prescriptions for H_2_ receptor antagonists declined more than those for proton pump inhibitors. Similarly, the reduction in Z-drug prescriptions exceeded that of benzodiazepines and newer drugs (i.e., ramelteon and suvorexant). Among analgesics, acetaminophen and nonsteroidal anti-inflammatory drugs (NSAIDs) were decreased by −43.3% and −47.8%, respectively, whereas opioids increased by 22.8%, particularly, patch formulation (149.0% increase).

Symptomatic drugs were more commonly initiated after the index date than preventive drugs (from approximately 12% for diuretics and hypnotics to 50.8% for analgesics).

### Analyses by cancer types

The prescription of drugs for the three major cancer types (gastric, colorectal, and lung) is presented in Table S1 for preventive drugs and Table S2 for symptomatic drugs. Regarding preventive drugs, the prevalence of iron was higher for gastric and colorectal cancers than for lung cancers. Patients with lung cancer had a higher prevalence of aspirin than those with gastric or colorectal cancer. Prescription patterns for symptomatic drugs were similar among the three major cancer types.

## Discussion

In this nationwide study in Japan, we observed that the prescription of various preventive and symptomatic drugs, except opioids, decreased after the initiation of comprehensive home medical care services for patients with advanced-stage cancer, corresponding to the last months of life.

The use of preventive drugs remains common among patients with cancer at the end-of-life stage ^(9), (11), (12)^. By contrast, the present study showed that the prescriptions of all preventive drugs decreased. Similar trends were observed among older adults in other recent studies on older adults receiving home medical care in Japan ^(18)^ and nursing home residents in Belgium ^(22)^. For statins, which showed the greatest decrease in prescriptions in the present study, accumulated evidence supports deprescribing among older adults with limited life expectancy ^(10), (13), (21), (23), (24)^, which might give perspectives to clinicians that using this medication might be inconsistent with the goals of care, and the potential harm might outweigh the benefits at this stage.

Moreover, the prescription of anticoagulants and antidiabetic drugs modestly decreased, which has also been reported in previous studies ^(17), (25), (26), (27)^. Physicians might decide to continue prescribing anticoagulants to reduce the risk of life-threatening events (e.g., stroke), which could have a great impact on patient survival and QOL ^(18)^, regardless of the cancer stage and prognosis. Established tools such as OncPal and STOPPFrail do not include anticoagulants in the list ^(13), (14)^, which might rationalize prescribing physicians not to deprescribe. More recently, an international Delphi study reached a consensus on recommendations regarding deprescribing of medications in the last 6 months of life that included oral anticoagulants in cases of high bleeding risk, suggesting that deprescribing of oral anticoagulants remains to be recognized as a major challenge as well in other countries ^(28)^. Regarding antidiabetic drugs, glucose control targets can be moderated in this population to prevent hypoglycemia ^(13), (14)^. The doses of antidiabetic drugs may have been reduced; however, these changes were not investigated in this study.

The prevalence of antidementia drugs in the present study was much lower than in our previous study ^(18)^. This was likely because the present study focused on patients with advanced-stage cancer, resulting in a younger population, even though both studies focused on older adults with limited life expectancy in home medical care settings. The effectiveness of antidementia drugs in patients with advanced dementia has been debated, and no clear consensus has been reached ^(29)^. The patients’ cognitive functions were unidentified in this study; however, the limited effectiveness of antidementia drugs might lead to deprescribing of these drugs.

The prevalence of drugs for osteoporosis greatly decreased in this study, consistent with a previous study ^(30)^. Moreover, bisphosphonates were less likely to benefit patients with a life expectancy of ≤12 months ^(31)^. Patients are required to sit or stand upright after taking the medication to avoid adverse gastrointestinal effects, which often becomes more difficult for older adults who receive home medical care approaching the end of life.

The prescriptions of most symptomatic drugs also decreased after the index date. The higher initiation rates for symptomatic drugs suggest that prescriptions were determined based on careful monitoring of symptoms at the end-of-life stage. The high and increased prescription of analgesics (predominantly opioids) was notable in this population to relieve pain, which is a common symptom in patients with end-of-life cancer. The increased use of opioids after the index date, along with the decreased prescriptions of acetaminophen and NSAIDs, may suggest an improvement in pain management for patients experiencing stronger and persistent pain as they approach the end of life. Nevertheless, compared with other countries ^(11), (32)^, older adults with cancer in Japan are less likely to receive opioids, as reported in hospitalized patients with dementia ^(33)^. Underprescription of opioid analgesics was observed in East Asian countries, including Japan; therefore, awareness of the appropriate use of analgesics should be increased in this region ^(34)^. The prevalence of laxatives and diuretics was similar to that in older adults with or without cancer who received home medical care ^(18)^; however, the prescription of acid suppressants was more common in this study population, suggesting the need for alleviating gastrointestinal symptoms caused by primary diseases or drugs. Controlling insomnia from a QOL perspective in patients with advanced-stage cancer is crucial ^(35)^. Hypnotics such as benzodiazepines require a certain amount of time to taper the dose ^(36)^, which might exceed their life expectancy; thus, deprescribing them in the end-of-life phase is not feasible. Ideally, physicians should conduct medication reviews at the earliest to ensure deprescribing benzodiazepines can be accomplished.

This study had some limitations. First, the study population was limited to only those who received the claims for the comprehensive home medical care services for patients with advanced-stage cancer; however, there might be some patients who did not receive the services despite similar clinical situations. Second, before the participants received the services, deprescribing might have been conducted already because the physicians considered their patients to be those with limited life expectancy, as was observed in our previous study where decreases were seen during the last year of life ^(18)^. Moreover, medication use after the index date might be underestimated because some drugs prescribed before the index date may have covered a supply that extended beyond it. Third, we could not identify the exact information about the deprescribing process. The reasons for deprescribing, such as response to adverse drug events or consideration of the time to benefit, are unclear. There is a lack of data regarding the use or prescriber awareness of the OncPal, STOPPFrail, and other guidelines or tools for appropriate medications in older adults ^(13), (14), (37)^. Apart from the guidelines and tools, swallowing dysfunction or deterioration of the condition might have appeared near the end of life, causing inability to swallow medications, which could be a strong incentive for deprescribing. Fourth, there might be certain situations that medication should continue to be prescribed. For example, although tight glucose control is not recommended for people near the end of life to avoid hypoglycemia, appropriate blood glucose level may reduce risks for infections and dehydration at the end-of-life stage. In addition, some medications should be gradually tapered over time (e.g., discontinuing medications one by one when multiple medications are involved or reducing the dosage incrementally). However, the claims database is not well-suited to capture such detailed changes based on individual clinical data; thus, further studies are needed to assess the appropriateness of deprescribing methods. Finally, there were some other factors that might have influenced the study results but were not well considered in this study. A nationwide survey showed that the involvement of pharmacists reduced inappropriate medication use in hospital settings ^(38)^; however, we did not identify who was involved in the decision-making process for our study participants. Further studies are needed to assess the contribution of multidisciplinary teams to medication reviews in home medical care settings. Similarly, advance care planning (ACP) has recently been incorporated into clinical practice in Japan. It is important to note that the Japanese culture values family-oriented decision-making, which requires a more complicated process in ACP. For older adults with limited life expectancy, discussions about discontinuing preventive medications should be conducted as part of this process with a multidisciplinary team, including physicians, pharmacists, nurses, and other healthcare professionals. Due to the potential for increased uptake of these activities in clinical practice, further studies are needed to verify our results using more recent and detailed data.

### Conclusions

This nationwide study in Japan showed that the prescriptions of most drugs, except opioids, decreased during the last months of life among older adults with advanced-stage cancer who received home medical care. Given that no established evidence or data on older adults with advanced-stage cancer in home medical settings are available, the results of this study will serve as a useful guide for appropriate pharmacotherapy at the end-of-life stage. A decrease was observed in the prescription of most drugs at the end of life; however, this study draws more attention to facilitating medication reviews and deprescribing of preventive drugs to avoid inappropriate polypharmacy.

## Article Information

### Author Contributions

Shota Hamada conceptualized, designed the study, collected data, and performed the analyses. All authors interpreted the data. Yukari Hattori and Shota Hamada drafted the manuscript. Masao Iwagami, Nobuo Sakata, Kiwami Kidana, Nanako Tamiya, Masahiro Akishita, and Takashi Yamanaka reviewed and revised the manuscript critically for important intellectual content. All authors have approved the final manuscript as submitted.

### Conflicts of Interest

Yukari Hattori, Shota Hamada, Kiwami Kidana, and Takashi Yamanaka belonged to an endowed chair funded by donations from Hakue Technology, PROUMED, Japan Bio Products, Towa Pharmaceutical, Yellow Eight, and Sugi Holdings. Masahiro Akishita received research funding from Eisai, Kracie Pharma, Mitsubishi-Tanabe Pharma, and lecture fees from Daiichi Sankyo.

### Ethical Consideration

This study was conducted in accordance with the ethical principles outlined in the Declaration of Helsinki. This study was approved by the ethical review boards of our institutions (Ethical Review Board at the Institute for Health Economics and Policy, Number H30-004; Research Ethics Committee of the Faculty of Medicine of the University of Tokyo, Number 2020309NI).

### Data Availability Statement

The datasets generated and/or analyzed during the current study are not publicly available because data extracted from the NDB is not permitted to use except for those who are authorized.

### Informed Consent

Informed consent from the study participants was waived as the data were anonymized before being provided to the researchers.

### Consent for Publication

Not applicable.

### Disclaimer

Masahiro Akishita is one of the Editors of JMA Journal and on the journal’s Editorial Staff. He was not involved in the editorial evaluation or decision to accept this article for publication at all.

## Supplement

Supplementary Material
