# Supplementary material for: Current Status of Drug Prescribing among Older Adults with Advanced-stage Cancer Receiving Home Medical Care in Japan: A Nationwide Study
Source: JMA J. 2025 Sep 26;8(4):1350–8. doi: 10.31662/jmaj.2025-0179 (PMC12598258; doi:10.31662/jmaj.2025-0179)
Supplement: Supplementary Material [file 2433-3298-8-4-1350-s001.pdf]

Table S1. Prescribing of Preventive Drugs According to Three Major Cancer Types.

Drugs	Gastric (N = 1,394)		Colorectal (N = 1,624)		Lung (N = 1,787)	
	Before (%)	After (%)	Before (%)	After (%)	Before (%)	After (%)
Antihypertensives	56.5	22.6	60.8	30.3	59.6	32.7
ACEI/ARB	31.8	8.6	36.1	13.1	34.0	14.7
β blockers	12.3	7.0	11.3	7.3	12.3	7.9
Calcium channel blockers	43.1	13.2	46.9	20.6	46.2	23.1
Diuretics[a]	6.2	1.9	6.5	2.4	6.0	2.6
Others	2.7	<1.0	3.7	1.4	3.1	1.6
Antiplatelets	20.4	8.3	17.9	7.8	26.1	13.5
Aspirin	12.1	4.4	11.9	4.9	17.4	8.4
P2Y12 inhibitors	7.7	3.6	6.3	2.9	9.1	4.3
Others	4.4	1.5	2.9	< 1.0	5.0	2.5
Oral anticoagulants	10.8	5.4	10.7	5.8	9.9	7.2
DOAC	8.9	4.4	7.8	4.2	7.6	5.5
Warfarin	2.5	1.0	3.1	1.7	2.6	1.7
Lipid-lowering drugs	23.3	3.8	23.0	5.5	27.6	7.1
Statins	20.3	3.2	20.4	4.6	24.7	6.6

Table S1. Prescribing of preventive drugs according to three major cancer types (cont'd)

Drugs	Gastric (N = 1,394)		Colorectal (N = 1,624)		Lung (N = 1,787)	
	Before (%)	After (%)	Before (%)	After (%)	Before (%)	After (%)
Antidiabetic drugs	17.4	7.5	17.8	10.0	17.8	11.0
Non-insulin	16.9	6.5	17.1	8.4	17.0	9.7
Insulin	2.9	1.7	4.1	2.7	4.3	2.9
Drugs for osteoporosis	17.6	5.2	17.1	4.2	18.3	6.0
Bisphosphonates	8.8	1.5	8.7	1.5	9.2	2.3
Vitamin D formulations	10.9	4.2	10.7	3.1	11.3	4.3
Antigout drugs	11.6	3.2	10.3	3.7	13.9	4.9
Antidementia drugs	10.7	5.6	10.9	6.2	9.3	5.3
Acetylcholinesterase inhibitors	8.4	4.0	8.7	4.6	7.9	7.9
Memantine	3.7	2.2	3.5	2.2	2.5	2.1
Calcium	2.2	<1.0	1.7	<1.0	1.5	<1.0
Iron	30.4	16.1	29.9	15.8	8.9	4.9
Vitamins (B, C or multivitamins)	19.2	5.3	18.9	5.4	23.1	6.7

ACEI: angiotensin-converting enzyme inhibitor; ARB: angiotensin II receptor blocker; DOAC: direct oral anticoagulant

[a]Diuretics as drugs other than thiazides and selective aldosterone blockers (e.g., loop diuretics and potassium-sparing diuretics).

Table S2. Prescribing of Symptomatic Drugs According to Three Major Cancer Types.

Drugs	Gastric (N = 1,394)		Colorectal (N = 1,624)		Lung (N = 1,787)	
	Before (%)	After (%)	Before (%)	After (%)	Before (%)	After (%)
Acid suppressants	69.1	53.4	60.2	50.1	65.2	56.7
Proton pump inhibitors	64.0	50.4	52.2	46.1	57.0	52.0
H2 receptor antagonists	13.1	3.5	13.7	5.0	14.7	6.0
Laxatives	64.1	50.2	70.2	55.4	65.9	64.2
Diuretics (non-antihypertensives)	29.9	26.7	29.2	29.0	23.1	22.5
Loop	28.0	24.7	26.8	26.9	21.1	20.7
MRA	13.1	12.0	12.4	12.9	7.8	7.1
Analgesics	73.4	66.7	78.0	71.8	78.7	76.4
Acetaminophen	49.3	28.8	51.2	35.0	52.3	35.3
NSAIDs	43.8	21.5	48.0	29.8	50.1	33.2
Opioids (oral and patch)	37.2	50.4	39.7	54.7	45.4	60.3
Opioids (oral)	34.4	42.0	37.4	46.8	43.6	55.4
Opioids (patch)	11.8	33.9	12.4	35.2	9.5	29.3
Hypnotics	31.9	25.8	33.3	28.3	35.3	31.7
Benzodiazepines	17.6	13.6	18.2	14.4	20.6	16.6
Z-drugs	15.6	10.6	15.8	11.5	16.0	11.4
Newer drugs	7.0	7.2	7.6	7.0	8.1	10.5

MRA: mineral corticoid receptor antagonist; NSAIDs: nonsteroidal anti-inflammatory drugs
